# Intrathecal kappa free light chain synthesis is associated with worse prognosis in relapsing–remitting multiple sclerosis

**DOI:** 10.1007/s00415-023-11817-9

**Published:** 2023-06-14

**Authors:** Igal Rosenstein, Markus Axelsson, Lenka Novakova, Clas Malmeström, Kaj Blennow, Henrik Zetterberg, Jan Lycke

**Affiliations:** 1 Department of Clinical Neuroscience, Institute of Neuroscience and Physiology at Sahlgrenska Academy, Sahlgrenska University Hospital, University of Gothenburg, Blå Stråket 7, 413 45 Gothenburg, Sweden; 2 Clinical Neurochemistry Laboratory, Sahlgrenska University Hospital, Mölndal, Sweden; 3 Department of Psychiatry and Neurochemistry, Institute of Neuroscience and Physiology, University of Gothenburg, Mölndal, Sweden; 4 UK Dementia Research Institute at UCL, London, UK; 5 Department of Neurodegenerative Disease, UCL Queen Square Institute of Neurology, London, UK; 6 Hong Kong Centre for Neurodegenerative Diseases, Hong Kong, China; 7 Wisconsin Alzheimer’s Disease Research Center, University of Wisconsin School of Medicine and Public Health, University of Wisconsin-Madison, Madison, WI USA

**Keywords:** Multiple sclerosis, Cerebrospinal fluid, Kappa free light chain index, Biomarkers, Prognosis

## Abstract

**Background:**

While kappa free light chain (KFLC) index has become a useful diagnostic biomarker in multiple sclerosis (MS), its prognostic properties are less explored. B cells play a crucial role in MS pathogenesis, but the impact from increased intrathecal production of immunoglobulins and KFLC remains to be determined. Recently, it has become evident that insidious worsening is not confined to progressive MS but is also common in relapsing–remitting MS (RRMS), a feature known as progression independent of relapse activity (PIRA).

**Methods:**

We retrospectively identified 131 patients with clinically isolated syndrome or early RRMS who had determined KFLC index as part of their diagnostic workup. Demographic and clinical data were extracted from the Swedish MS registry. Associations of baseline KFLC index with evidence of disease activity (EDA) and PIRA were investigated in multivariable cox proportional hazards regression models.

**Results:**

KFLC index was significantly higher in PIRA (median 148.5, interquartile range [IQR] 106.9–253.5) compared with non-PIRA (78.26, IQR 28.93–186.5, p = 0.009). In a multivariable cox regression model adjusted for confounders, KFLC index emerged as an independent risk factor for PIRA (adjusted hazard ratio [aHR] 1.005, 95% confidence interval [CI] 1.002–1.008, p = 0.002). Dichotomized by the cut-off value KFLC index > 100, patients with KFLC index > 100 had an almost fourfold increase in the risk for developing PIRA. KFLC index was also predictive of evidence of disease activity during follow-up.

**Conclusions:**

Our data indicate that high KFLC index at baseline is predictive of PIRA, EDA-3, and overall worse prognosis in MS.

## Introduction

In recent years, kappa free light chain (KFLC) index, a measure of intrathecal free kappa chains, has become a useful diagnostic biomarker in multiple sclerosis (MS) [1–4]. However, its prognostic ability is less explored. Several studies have previously shown that high intrathecal KFLC synthesis predicts the conversion from clinically isolated syndrome (CIS) to clinically definitive MS (CDMS) or a second demyelinating event [5–10]. In addition, high KFLC index has been shown to predict Expanded Disability Status Scale (EDSS) ≥ 3, therapy escalation, and cognitive decline [11, 12].

More recently, it has been increasingly recognized that deterioration in disability can occur early in the course of relapsing–remitting MS (RRMS), often in the absence of clinical relapses [13–15]. This type of clinical worsening, termed progression independent of relapse activity (PIRA), is distinct from relapse-associated worsening (RAW), in that it is often associated with an increased risk of severe disability [16], higher rates of cortical atrophy [17], and entails overall worse prognosis, especially when it occurs early in the disease [16]. It is therefore vital to identify patients with RRMS who are at increased risk of PIRA early, as highly effective disease-modifying therapies (DMT) may reduce such insidious deterioration [13]. Several demographic and clinical factors, such as older age, longer disease duration at baseline (BL), and a relapsing–remitting disease course at onset, have been associated with a higher risk of PIRA [14].

Our objective was to investigate whether intrathecal KFLC synthesis, determined at the diagnostic investigation of RRMS, may predict a higher risk for disability worsening in both patients with (RAW) and without relapse (PIRA). We also examined if KFLC index predicts evidence of disease activity (EDA), including activity determined on magnetic resonance imaging (MRI).

## Materials and methods

This was a retrospective cohort study of prospectively obtained CSF and clinical data collected from routine clinical investigations and follow-up of CIS and MS patients at the MS centre, Sahlgrenska University Hospital, Gothenburg, Sweden. All patients fulfilled the 2017 revised McDonald criteria for CIS or RRMS [18]. CSF sampling took place between 12-07-2013 and 30-12-2020. We excluded patients who were classified as having progressive MS at the time of sampling (n = 42). In addition, 11 patients were lost to follow-up after the initial investigation and were thus excluded. Other inclusion criteria were a minimum follow-up time of at least 24 months and inclusion in the Swedish MS registry (SMSreg, http://www.msreg.net) [19]. Demographical and clinical data were extracted from the SMSreg. Disability was determined with the Expanded Disability Status Scale (EDSS) [20] at BL and again in cases where patients had clinical relapses during follow-up at re-baseline (RBL) (see below), and thereafter at least annually. MRI of the brain and spinal cord without and with gadolinium contrast was performed on 1.5 or 3.0 T machines, according to Swedish radiological guidelines [21]. MRIs were obtained at BL, about 6 months after BL, and thereafter at least annually, and in addition, on the discretion of the treating physician in cases of suspected relapse. Data on T1 contrast-enhancing lesions (CEL) and new/newly enlarging T2-weighted (T2W)-lesions at follow-up, as well as demographic data and information about clinical relapses, disability, MRIs and exposure to DMTs were retrieved from the SMSreg and patients’ electronic journals.

### Study endpoints

The study population was first dichotomized according to whether patients exhibited evidence of disease activity (EDA) according to the No Evidence of Disease Activity (NEDA)-3 criteria [22]. EDA-3 was defined as the occurrence of either clinical relapses, and/or confirmed disability worsening (CDW) that was sustained for at least 6 months, and new T1 gadolinium-enhanced lesions/new/newly enlarging T2W lesions. A clinical relapse was defined as neurological signs and symptoms lasting at least 24 h and that could not be explained by another cause [18]. Patients were thus dichotomized to those who maintained NEDA-3 status during the whole follow-up period and those who demonstrated EDA-3.

Thereafter, we chose to focus on those individuals in the EDA-3 group who experienced CDW unrelated to preceding relapses (i.e., had PIRA). The main endpoint of interest in the study was hence a first PIRA event, which was defined according to previous publications [13, 14, 16]. A first PIRA event was defined as experiencing CDW in the EDSS at 6 months during a period not preceded by a relapse (at least 3 months before the PIRA event or 6 months after the first demyelinating event). The first EDSS score obtained between 6 and 12 months after the first demyelinating attack or alternatively 3 months after any other attack was referred to as the BL EDSS score and RBL EDSS score, respectively. As previously described [13], no RBL EDSS score could be lower than the first recorded (BL) EDSS score. CDW was defined as an increase in the EDSS score of 1.5, 1.0, or 0.5 if the BL/RBL EDSS score was 0, 1.0 to 5.0, or greater than 5.0, respectively. The date of PIRA was the date of CDW confirmation. According to this definition, individuals included in the study were stratified into two groups, non-PIRA and PIRA.

### Analyses of intrathecal immunoglobulin synthesis

Paired CSF and serum samples were acquired and consecutively analysed during the routine diagnostic work-up. Concentrations of KFLC in serum and CSF were measured using the N Latex FLC kappa kit, on an Atellica NEPH 630 instrument (Siemens), following the instructions by the manufacturer. The KFLC index was calculated using the equation [(CSF KFLC/serum KFLC)/(CSF albumin/serum albumin)]. CSF and serum albumin concentrations were measured using the ALBT2 Reagent cassette on a cobas c module instrument (Roche). The CSF/serum albumin ratio was calculated as [CSF albumin (mg/L)/serum albumin (g/L)]. CSF and serum IgG concentrations were measured using the IGG-2 reagent cassette on a cobas c module instrument (Roche). The IgG index was calculated as [CSF IgG (mg/L)/serum IgG (g/L)/CSF albumin (mg/L)/serum albumin (g/L)].

CSF-specific IgG oligoclonal bands (OCBs) were determined using an in-house isoelectric focusing (IEF) method on 7.7% polyacrylamide gels with subsequent silver staining. Matched patient serum and CSF samples were run on adjacent lanes, and CSF-specific IgG OCBs were defined as extra bands in the gamma-zone, which were not present in the corresponding serum sample. For quality control, a positive CSF sample with known CSF-specific OCBs was run on each gel. A cut-off value of IgG OCB ≥ 2 was considered positive. Board-certified laboratory technicians, who were blinded to the clinical status, using strict procedures for quality control and run-approval, performed the analyses. All analyses were performed at the Sahlgrenska Neurochemistry Laboratory in Mölndal, Sweden.

### Determination of other CSF biomarkers

As part of the diagnostic routine for MS investigations we also measured neurofilament light (NfL, n = 131), glial fibrillary acidic protein (GFAP) (n = 131), and tau (n = 125) concentrations in CSF. CSF NfL concentration was measured using a sensitive sandwich enzyme-linked immunosorbent assay (ELISA; NF-light® ELISA kit; UmanDiagnostics AB, Umeå, Sweden; Catalog # 10–7001 CE) as previously described [23], or with an in-house ELISA method as described previously in detail [24]. Comparison of the in-house ELISA and the UmanDiagnostics ELISAs showed CSF NfL concentrations in the same range and a strong linear correlation between CSF NfL values [24]. CSF GFAP concentration was measured by ELISA, as previously described [25]. CSF tau concentration (INNOTEST® hTAU Ag; Product # 81,572) was measured by ELISA, as previously described [26].

### Statistical analysis

Data are presented as mean ± SD or as median and interquartile range (IQR), as appropriate. Data distribution was assessed with the Shapiro-Wilks test. The Mann–Whitney U test, unpaired T test, χ^2^ test, and Fisher’s exact test were used for group comparisons as appropriate. The Mann–Whitney U test was used for all comparisons of CSF biomarkers in EDA-3 vs NEDA-3 and non-PIRA vs PIRA. EDSS values at BL/RBL and follow-up stratified by non-PIRA/PIRA were compared with the Wilcoxon matched-pairs signed rank test, and the p value threshold for multiple comparisons was set with the Holm-Šídák method. Next, based on the results of CSF biomarker comparisons, we select CSF KFLC, KFLC index and CSF tau for further analysis in univariable and multivariable cox proportional hazards regression models. As described above, two endpoints were used as time-dependent variables, EDA-3, and PIRA. Cox proportional hazards regression models were performed and the adjusted hazard ratios (aHR) along with corresponding 95% confidence intervals (CI) were calculated. Time to EDA-3 and PIRA were investigated with Kaplan–Meier survival analyses with corresponding logrank tests. For visualization in Kaplan–Meier curves with KFLC index as a predictor variable and based on previous work on the prognostic value of KFLC index, we stratified the cohort according to the cut-off value KFLC index > 100 [9, 12]. Based on previous investigations on prognostic factors, we chose to adjust the models for the following potential confounding covariates: age at the time of CSF sampling, sex, total follow-up time, time from sampling to DMT start, disease course at onset (CIS/RRMS), onset EDSS in the case of EDA-3 (i.e. the first EDSS score recorded at the time of sampling), or BL/RBL EDSS as defined above in the case of PIRA, and brain MRI characteristics (T2W lesions and BL CEL). In the model with EDA-3 as a dependent variable, we adjusted for exposure to high-efficacy DMT from onset. In the model with PIRA as a dependent variable, we adjusted for treatment strategy during follow-up (three categories: 1. patients who received first-line therapy during the whole follow-up; 2. patients who escalated therapy during the follow-up; and 3. patients who initiated high-efficacy DMT at baseline). Natalizumab, anti-CD20 therapies (rituximab or ocrelizumab), alemtuzumab, and autologous hematopoietic stem cell transplantation (AHCST) were classified as high-efficacy DMT, whereas sphingosine-1-phosphate receptor modulators (fingolimod, ponesimod, siponimod or ozanimod), cladribine tablets, teriflunomide, dimethyl fumarate, platform therapies, or no DMT, were grouped as low/moderate efficacy (first-line) DMT. We tested KFLC index > 100 as a binary categorical variable separately. Results of the other confounding covariates are presented only for the model including KFLC index as a continuous variable, as they did not substantially differ in the other models. Due to the exploratory nature of the study, no correction for multiple comparisons in the survival analyses was made. Statistical significance was assumed at p < 0.05. All statistical analyses and figures were performed/created with IBM SPSS version 28.0.1.0 (Armonk, NY: IBM Corp. 2011) and GraphPad prism version 9.1.0.

### Ethical considerations

All patients included in this study had given informed consent to be registered in the SMSreg. All individual data from the different sources were made anonymous to the authors by the replacement of the personal identity numbers by unique number codes for use in the present study. The study has been approved by the Swedish Ethical Review Agency (Dnr: 2020-06851).

### Data availability

Anonymized data, not published in the article, will be shared on reasonable request from a qualified investigator.

## Results

**Fig. 1:**
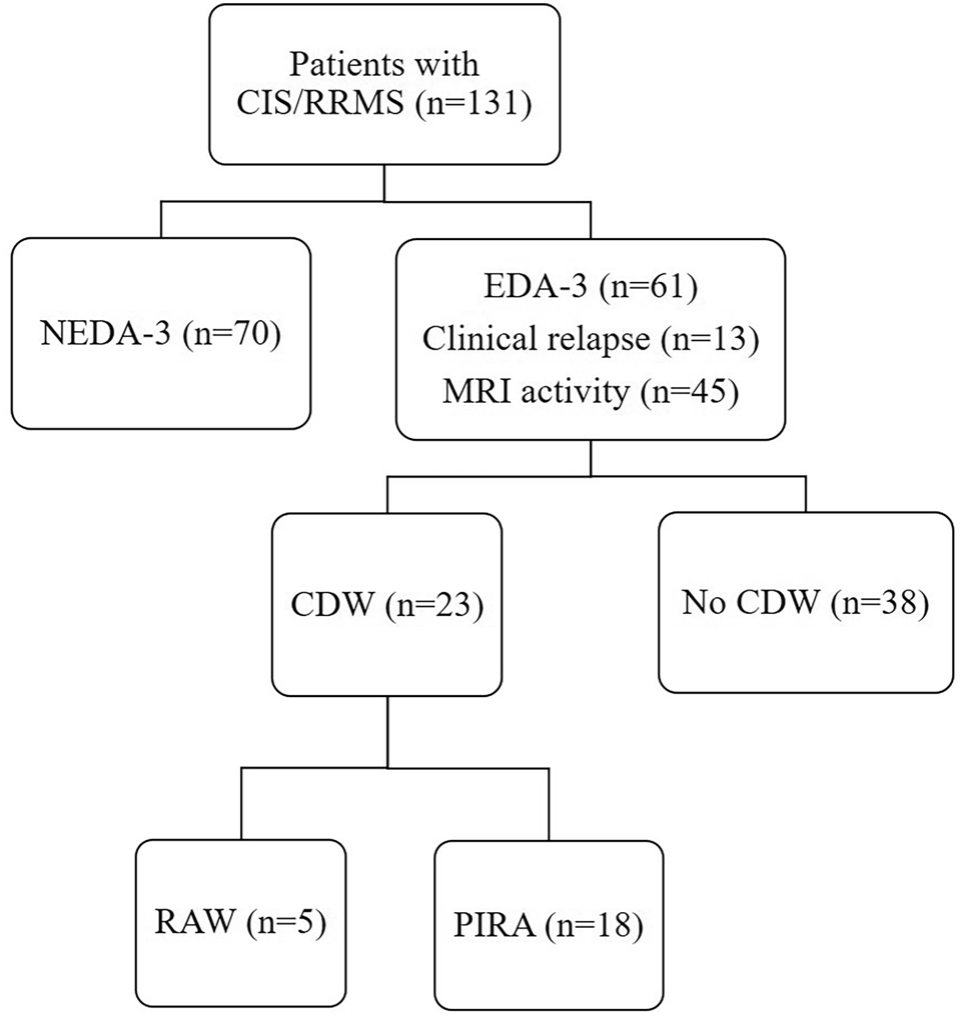
Hierarchy chart showing the classification of RRMS patients based on EDA-3/NEDA-3 status and CDW. *CIS* clinically isolated syndrome, *RRMS* relapsing–remitting multiple sclerosis, *NEDA* no evidence of disease activity, *EDA* evidence of disease activity, *MRI* magnetic resonance imaging, *CDW* confirmed disability worsening, *PIRA* progression independent of relapse activity, *RAW* relapse-associated worsening

The study population was comprised of 131 individuals of which 17 (13%) were classified as CIS at diagnosis and the rest as RRMS (87%). All patients initially designated as CIS but two eventually converted to clinically definitive MS during the study follow-up. The majority (69.5%) were female, with a median (IQR) age at symptom onset of 34 years (28–42) (Table 1). Median (IQR) disease duration prior to presentation was 5 (1–22) months. The total median (IQR) follow-up time for the whole cohort was 41 (33–72) months. Most individuals (n = 75, 57.3%) were assigned a high-efficacy DMT from disease onset. Overall, 15 patients (11.5%) escalated therapy during follow-up, nine with CIS who converted to CDMS and escalated from no DMT to DMT, and six with RRMS who escalated from first-line to high-efficacy DMT. BL KFLC index did not significantly differ between the different treatment groups (p = 0.23). Sixty-one (46.6%) individuals presented with an EDA-3 event according to the definition above within the total follow-up time. Of these, 23 had CDW, of which 18 (13.7%) individuals presented with at least one PIRA event during follow-up (Fig. 1). The majority of PIRA events (17/18, 94.4%) were classified as early PIRA (i.e. PIRA within 5 years from onset). Demographical and clinical characteristics of the study population are presented in Table 1.

**Table 1:** Clinical and demographical characteristics of CIS/RRMS patients included in the study and dichotomized by EDA/NEDA and non-PIRA/PIRA status at follow-up

Variable	Total CIS/RRMS patients (n = 131)	NEDA (n = 70)	EDA (n = 61)	*p*-value	Non-PIRA (n = 113)	PIRA (n = 18)	*p*-value
Age, median (IQR)	34 (28–42)	35.5 (27.7–44)	32 (28–41)	0.56^**a**^	34 (28–41)	34 (28–44.5)	0.7^a^
Sex (Female), n (%)	91 (69.5)	51 (72.9)	40 (65.6)	0.45^b^	80 (70.8)	11 (61.1)	0.42^b^
Disease duration from symptom onset to sampling, m, mean ± SD	25.8 ± 51.1	32.5 ± 65	18.1 ± 37.1	0.13^c^	25.1 ± 53.5	30.4 ± 59.6	0.7^c^
Total follow-up time from first to last visit, m, median (IQR)	41 (33–72)	40 (32.5–48)	47 (33–84)	**0.022**^**a**^	41 (31 -72)	48.5 (37.5–100.5)	0.0504^a^
Onset EDSS, median (IQR)	2 (1–3)	2 (1–3)	2 (1–2.75)	0.72^**a**^	2 (1–2.75)	2 (1–3)	0.64^**a**^
BL/RBL EDSS, median (IQR)	2 (1–3)	2 (1.4–3)	1.5 (1–2.5)	0.1^**a**^	2 (1.3–3)	1 (1–1.5)	**0.004**^**a**^
Disease course at sampling, n (%)							
CIS	17 (13)	2 (2.9)	15 (24.6)	** < 0.001**^**b**^	16 (14.2)	1 (5.6)	0.46^b^
RRMS	114 (87)	68 (97.1)	46 (75.4)		97 (85.8)	17 (94.4)	
BL T2W lesions, n (%)				0.86^b^			0.13^b^
< 10	61 (46.6)	32 (45.7)	29 (47.5)		56 (49.6)	5 (27.8)	
≥ 10	70 (53.4)	38 (54.3)	32 (52.5)		57 (50.4)	13 (72.2)	
CEL, n (%)	70 (53.4)	36 (51.4)	34 (55.7)	0.73^b^	62 (54.9)	8 (44.4)	0.45^b^
IgG OCBs ≥ 2, n (%)	128 (97.7)	68 (97.1)	60 (98.4)	1.0^b^	110 (97.3)	18 (100)	1.0^b^
Qalb (× 10^3^), median (IQR)	3.9 (3.1–5.5)	3.9 (2.9–5.2)	3.9 (3.2–5.9)	0.76^a^	3.9 (3.2–5.5)	4.15 (2.6–5.9)	0.91^a^
CSF KFLC mg/L, median (IQR)	4.8 (2.1–9.9)	3.7 (1.6–8.5)	6.17 (3.5–13)	**0.003**^**a**^	4.1 (1.8–9.9)	8.8 (4.6–19.3)	**0.008**^**a**^
Serum KFLC mg/L, median (IQR)	13 (10.4–16.2)	13.5 (10.6–17.1)	12.9 (10.3–14.8)	0.31^a^	13 (10.4–16.1)	12.7 (10.4–16.3)	0.88^a^
KFLC index, median (IQR)	96.1 (30.1–187)	66.3 (25.3–177.2)	121.4 (56.7–191.4)	**0.015**^**a**^	78.3 (28.9–186.5)	148.5 (106.9–253.5)	**0.009**^**a**^
IgG index, median (IQR)	0.89 (0.68–1.34)	0.81 (0.64–1.3)	0.93 (0.75–1.42)	0.083^a^	0.86 (0.65–1.31)	1.12 (0.91–1.81)	**0.016**^**a**^
CSF NfL ng/L, median (IQR)	970 (530–2160)	975 (575–2163)	930 (505–2185)	0.92^a^	970 (550–2040)	980 (330–2498)	0.99^a^
Tau ng/L, median (IQR)	198 (163.5–268)	194 (158–232)	224.5 (172.8–299)	**0.048**^**a**^	198 (163–267)	215 (163–324.5)	0.58^a^
CSF GFAP ng/L, median (IQR)	330 (230–440)	320 (227.5–442.5)	350 (245–440)	0.42^a^	330 (230–420)	370 (257.5–465)	0.39^a^
Time from sampling to DMT start, d, median (IQR)	34 (19–100)	31 (19–49)	55 (19–196.5)	**0.018**^**a**^	35 (20–105.3)	27 (0–59)	**0.03**^**a**^
Treatment strategy, n (%)				** < 0.001**^**b**^			0.3^b^
First-line	41 (31.3)	23 (32.9)	18 (29.5)		36 (31.9)	5 (27.8)	
Escalation	15 (11.5)	1 (1.4)	14 (23)		11 (9.7)	4 (22.2)	
he-DMT	75 (57.3)	46 (65.7)	29 (47.5)		66 (58.4)	9 (50)	
Dominant DMT during the whole follow-up, n (%):				0.08^b^			0.13^b^
No DMT	3 (2.3)	3 (4.3)	0 (0)		3 (2.7)	0 (0)	
Dimethyl fumarate	27 (20.6)	9 (12.9)	18 (29.5)		24 (21.2)	3 (16.7)	
Teriflunomide	3 (2.3)	2 (2.9)	1 (1.6)		2 (1.8)	1 (5.6)	
Fingolimod	6 (4.6)	1 (1.4)	5 (8.2)		3 (2.7)	3 (16.7)	
Natalizumab	32 (24.4)	20 (28.6)	12 (19.7)		31 (27.4)	1 (5.6)	
Rituximab	41 (31.3)	25 (35.7)	16 (26.2)		34 (30.1)	7 (38.9)	
Cladribine	13 (9.9)	8 (11.4)	5 (8.2)		11 (9.7)	2 (11.1)	
Alemtuzumab	4 (3.1)	1 (1.4)	3 (4.9)		3 (2.7)	1 (5.6)	
AHSCT	2 (1.5)	1 (1.4)	1 (1.6)		2 (1.8)	0 (0)	

Bold text indicates *p* values < 0.05

*CIS* clinically isolated syndrome, *RRMS* relapsing–remitting multiple sclerosis, *NEDA* no evidence of disease activity, *EDA* evidence of disease activity, *PIRA* progression independent of relapse activity, *SD* standard deviation, *BL* baseline, *RBL* re-baseline, *EDSS* expanded disability status scale, *IQR* interquartile range, *MRI* magnetic resonance imaging, *IgG* immunoglobulin G, *OCB* oligoclonal bands, *KFLC* kappa free light chain, *Qalb* albumin quotient, *CSF* cerebrospinal fluid, *NfL* neurofilament light, *GFAP* glial fibrillary acidic protein, *CEL* contrast-enhancing lesion, *he* high-efficacy, *DMT* disease modifying therapy, *AHSCT* autologous hematopoietic stem cell transplantation

Data are shown as median and interquartile range unless otherwise specified

^a^Mann–Whitney U test

^b^Fisher’s exact test or Pearson chi-square test

^c^Unpaired T-test

### Intrathecal KFLC synthesis is higher in EDA-3 vs NEDA-3 and in PIRA vs non-PIRA

**Fig. 2:**
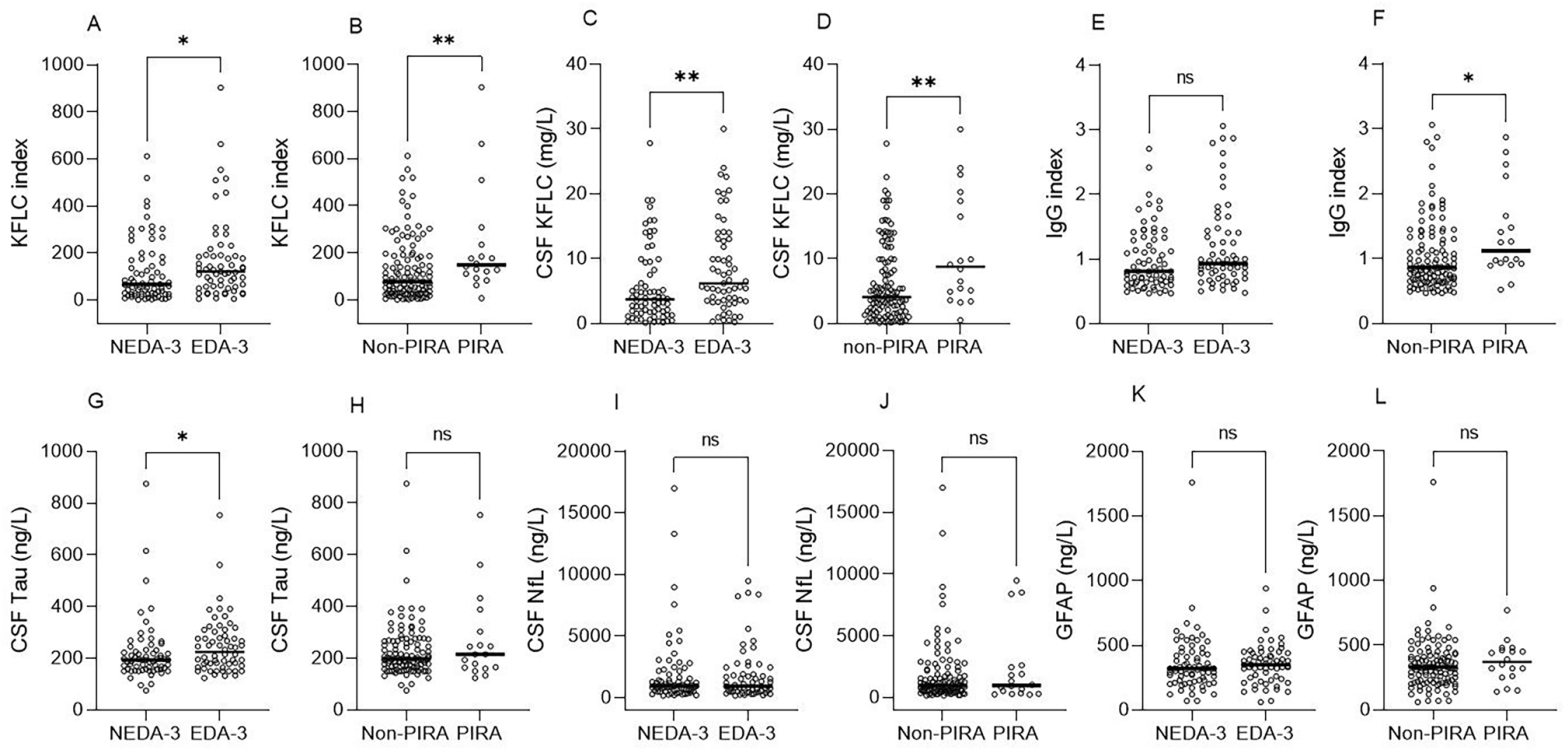
Scatter plots showing CSF biomarker concentrations at baseline in patients who remained NEDA-3 along the whole follow-up compared to patients who exhibited EDA-3, and in patients who remained non-PIRA compared to those who experienced a PIRA event during follow-up. Biomarker concentrations at baseline were compared with the the Mann–Whitney U test. Bar represents median. *KFLC* kappa free light chain, *CSF* cerebrospinal fluid, *Ig* immunoglobulin, *NfL* neurofilament light, *GFAP* glial fibrillary acidic protein, *NEDA* no evidence of disease activity, *EDA* evidence of disease activity, *PIRA* progression independent of relapse activity; ns = non-significant; * p < 0.05; ** p < 0.01

Patients who presented with an EDA-3 event during follow-up had higher KFLC index at BL compared with patients who remained stable (median KFLC index 121.4 [IQR 56.7 – 191.4] vs. 66.3 [25.3–177.2], p = 0.015) (Table 1, Fig. 2A). Likewise, patients who presented with a PIRA event during follow-up had higher median KFLC index at BL (148.5 [106.9–253.5] vs. 78.3 [28.9–186.5], p = 0.009) (Fig. 2B). Furthermore, CSF levels of KFLC were significantly higher in EDA-3 vs. NEDA-3 and PIRA vs. non-PIRA (Fig. 2C, D). The IgG index was also noted to be significantly higher in PIRA vs. non-PIRA but not in EDA-3 vs. NEDA-3 (Fig. 2E, F). The correlation between KFLC index and IgG index was very high (Spearman’s rho = 0.85, p < 0.001), and we therefore opted to focus on KFLC index and not the IgG index hereafter. Patients in the EDA-3 group had slightly but significantly higher CSF tau levels compared with the NEDA-3 group (Fig. 2G). No differences in CSF NfL and CSF GFAP concentrations between any of the groups were observed (Figs. 2I–L).

### Intrathecal KFLC synthesis is an independent risk factor for EDA-3

Most cases achieved EDA-3 due to MRI signs of disease activity (n = 45, 73.8%). In only 13 patients, EDA-3 was due to a clinical relapse. In univariable cox regression models, both CSF KFLC concentrations and the KFLC index were associated with increased hazard of EDA-3 (HR 1.046, 95% CI 1.012–1.081, p = 0.008 For CSF KFLC; HR 1.001, 95% CI 1.00006–1.003, p = 0.041 for KFLC index) (Table 2). Furthermore, both RRMS disease course and exposure to high-efficacy DMT were protective (HR 0.35, 95% CI 0.19–0.63, p < 0.001 for RRMS; HR 0.54, 95% CI 0.32–0.91, p = 0.02). In contrast, other demographical and clinical factors such as age, sex, BL EDSS and MRI measures were not associated with increased risk of EDA-3. As CSF tau levels were slightly but significantly increased in EDA-3 compared with NEDA-3, we opted to include CSF tau as well in the cox regression model. However, in univariable and multivariable analyses, CSF tau was not predictive of a higher EDA-3 risk.

**Table 2:** Univariable and multivariable cox proportional hazard regression models for intrathecal biomarkers measured at diagnosis as well as covariates and prediction of EDA-3 and PIRA

	EDA-3			PIRA		
	HR	95%CI	*p*-value	HR	95%CI	*p*-value
Univariable						
KFLC index^a^	1.001	1.00006–1.003	**0.041**	1.003	1.001–1.006	**0.003**
KFLC index > 100^b^	1.99	1.19–3.34	**0.008**	4.6	1.5–14.07	**0.007**
CSF KFLC^a^	1.046	1.012–1.081	**0.008**	1.087	1.027–1.15	**0.004**
CSF Tau^a^	1.001	0.99–1.003	0.387	1.002	0.99–1.005	0.224
Disease duration	0.99	0.98–1.002	0.172	1.002	0.99–1.010	0.642
Total follw-up time	1.007	0.99–1.016	0.17	1.002	0.99–1.01	0.64
Time from sampling to DMT start	1.001	1–1.002	0.037	0.99	0.98–1.002	0.14
Age	0.99	0.96–1.014	0.39	1.008	0.96–1.05	0.73
Sex (ref. F)	1.2	0.71– 2.05	0.48	1.51	0.58–3.9	0.39
Disease course (ref. CIS)	0.35	0.19–0.63	** < 0.001**	2.98	0.39–22.47	0.29
Onset EDSS	0.98	0.79–1.21	0.82	–	–	**–**
BL/RBL EDSS	–	–	–	0.61	0.4–0.93	**0.02**
BL T2W lesions (ref. < 10)	0.94	0.567–1.551	0.802	2.4	0.85–6.7	0.099
BL CEL	1.11	0.67–1.84	0.67	0.7	0.27–1.75	0.43
Exposure to he-DMT from onset	0.54	0.32–0.91	**0.02**	–	–	–
Treatment strategy						
First-line	–	–	**–**	Ref	–	–
Escalation	–	–	**–**	1.88	0.5–7.1	0.35
he-DMT	–	–	**–**	0.9	0.3–2.7	0.86
Multivariable						
KFLC index^a^	1.002	1.001–1.004	**0.007**	1.005	1.002–1.008	**0.002**
KFLC index > 100^b^	2.1	1.25–3.61	**0.005**	3.7	1.101–12.3	**0.03**
CSF KFLC^a^	1.053	1.016–1.09	**0.005**	1.08	1.006–1.16	**0.033**
CSF Tau^a^	1.001	0.99–1.003	0.47	1.003	0.99–1.006	0.099
Disease duration	0.996	0.99–1.003	0.27	1.001	0.99–1.013	0.86
Total follw-up time	1.006	0.996–1.016	0.27	1.01	0.99–1.031	0.362
Time from sampling to DMT start	0.999	0.998–1.001	0.6	0.99	0.98–1.001	0.072
Age	0.99	0.97–1.027	0.882	1.002	0.95–1.06	0.94
Sex (ref. F)	1.61	0.9–2.87	0.1	3.24	1.001–10.49	0.05
Disease course (ref. CIS)	0.235	0.09–0.61	**0.003**	0.947	0.034–26.16	0.97
Onset EDSS	1.101	0.877–1.34	0.4	–	–	**–**
BL/RBL EDSS	–	–	–	0.624	0.344–1.13	0.12
BL T2W lesions (ref. < 10)	1.26	0.68–2.3	0.46	3.64	0.97–13.6	0.054
BL CEL	1.41	0.82–2.44	0.21	0.63	0.23–2.422	0.63
Exposure to he-DMT from onset	0.521	0.28–0.96	**0.036**	–	–	–
Treatment strategy						
First-line	–	–	–	Ref	–	–
Escalation	–	–	–	4.48	0.66–30.25	0.124
he-DMT	–	–	–	0.6	0.16–2.24	0.45

Bold text indicates *p* values < 0.05

*EDA* evidence of disease activity, *HR* hazard ratio, *CI* confidence interval, *CSF* cerebrospinal fluid, *KFLC* kappa free light chain, *Ig* immunoglobulin, *he* high-efficacy, *DMT* disease modifying therapy, *BL* baseline, *RBL* re-baseline, *EDSS* expanded disability status scale, *T2W* T2-weighted, *CEL* contrast-enhancing lesion

^a^Continuous variable

^b^Categorical binary variable

In a multivariable cox regression model, KFLC index as a continuous variable was an independent predictor of EDA-3 status at follow-up (aHR 1.002, 95% CI 1.001–1.004, p = 0.007) (Table 2). In addition, CSF KFLC also emerged as an independent predictor of EDA-3 status (aHR 1.053, 95% CI 1.016–1.09, p = 0.005). RRMS disease course at onset was independently associated with a protective effect against EDA-3 (aHR 0.235, 95% CI 0.09–0.61, p = 0.003). Likewise, the protective effect of exposure to high-efficacy DMT from disease onset was preserved (aHR 0.521, 95% CI 0.28–0.96, p = 0.036). Similar to the univariable models, other demographic and clinical factors were not predictive of EDA-3, as shown in Table 2.

**Fig. 3:**
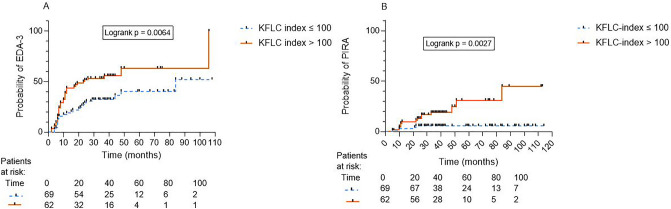
Kaplan–Meier survival curves showing time to (A) evidence of disease activity (EDA) -3 and, (B) progression independent of relapse activity (PIRA) and results of the logrank test as stratified by baseline KFLC index > 100 in patients with relapsing–remitting multiple sclerosis. *EDA* evidence of disease activity, *KFLC* kappa free light chain, *PIRA* progression independent of relapse activity

As a next step, to investigate the time to EDA-3 in a Kaplan–Meier survival analysis, we opted to test KFLC index as a binary categorical variable based on the prognostic cut-off value KFLC index > 100. In univariable and multivariable cox regression models, KFLC index > 100 was associated with increased risk of EDA-3 (multivariable model—aHR 2.1, 95% CI 1.25–3.61, p = 0.005). The median time to EDA-3 for KFLC index > 100 was 23.5 months compared with 84 months in patients with KFLC index ≤ 100 (logrank p = 0.0064) (Fig. 3A).

### High KFLC index is an independent predictor of PIRA

In patients with EDA-3, 23 patients exhibited CDW, of which five were classified as RAW, and 18 as PIRA (Fig. 1). In the PIRA subgroup, 4/18 (22.2%) had new T2W lesions on MRI during follow-up. In non-PIRA, BL/RBL EDSS were significantly higher compared with last follow-up (p < 0.001), while in PIRA, EDSS at censoring was significantly higher compared with BL/RBL EDSS (p < 0.001).

In a univariable cox regression model, KFLC index was associated with a higher risk of PIRA (HR 1.003, 95% CI 1.001–1.006, p = 0.003 for KFLC index) (Table 2). In addition, a lower BL EDSS was associated with PIRA (HR 0.61, 95% CI 0.4–0.93, p = 0.02). None of the other demographical and clinical factors demonstrated a significant association with PIRA.

In a multivariable cox regression model, KFLC index emerged as an independent risk factor for PIRA at follow-up (aHR 1.005, 95% CI 1.002–1.008, p = 0.002) (Table 2). Likewise, CSF KFLC levels were also independently associated with PIRA at follow-up (aHR 1.08, 95% CI 1.006–1.16, p = 0.033). The association of low BL EDSS diminished in the multivariable model. Notably, exposure to high-efficacy DMT from onset did not show a significant protective effect against the risk of PIRA (aHR 0.6, 95% CI 0.16–2.24, p = 0.45).

Next, similarly to the analysis of time to EDA-3, we opted to test KFLC index > 100 as a binary categorical variable to investigate time to PIRA. In a multivariable cox regression, KFLC index > 100 was an independent predictor of PIRA (aHR 3.7, 95% CI 1.101–12.3, p = 0.03). The median time to PIRA in both non-PIRA and PIRA remained undefined (logrank p = 0.0027) (Fig. 3B).

## Discussion

In this study, we investigated the prognostic utility of KFLC index in predicting EDA-3 status and PIRA in a cohort of CIS and early RRMS patients. We demonstrate that high levels of CSF KFLC as well as high KFLC index at BL are independent risk factors for EDA-3 status and PIRA during a mean follow-up of about four years. For every one unit incremental increase in KFLC index, higher risks for EDA-3 (0.2%) and PIRA (0.5%) were observed, and patients who had BL KFLC index > 100 had 2.1- and 3.7-fold increase in the risk of exhibiting EDA-3 and PIRA, respectively. None of the other CSF biomarkers that were included in our analysis showed similar prognostic ability.

Only a few studies have so far investigated the prognostic utility of KFLC index in MS [6–10, 12, 27, 28]. With one exception [27], they show that KFLC index can predict conversion from CIS to CDMS [6–10]. However, most of these study populations consisted mainly of CIS patients in which the majority were untreated with DMTs. Furthermore, only a few studies have attempted to explore the association of intrathecal KFLC synthesis with long-term disease worsening. While KFLC index correlates with a shorter time to EDSS progression [8] and predicts multiple sclerosis severity score (MSSS) [28], the ability to predict EDSS ≥ 3 has been inconclusive [9, 11]. Moreover, intrathecal KFLC concentrations have been shown to associate with brain atrophy [29]. Finally, we recently demonstrated that high KFLC index was associated with cognitive decline as determined by reduced SDMT scores by ≥ 8 points at follow-up [12].

Notably, CSF KFLC concentrations demonstrated comparable results to the KFLC index, and in the univariable analysis with EDA-3 as the endpoint, the effect size for CSF KFLC was considerably larger compared to the KFLC index. Arguably, the KFLC index is better scientifically justified and is often preferred to isolated CSF KFLC concentration measurements as it provides a correction for blood–brain barrier disruption. Although blood–brain barrier function rarely affects the interpretation of intrathecal KFLC synthesis in MS, most laboratories obtain paired serum and CSF samples for the diagnostic investigation of a suspected MS case. A single stand-alone CSF KFLC concentration assay is less expensive than running four assays across different laboratory platforms, providing some support for CSF KFLC concentration assessments when serum samples are not available.

In the present study, we could demonstrate that KFLC index had a prognostic significance even in a real-world cohort. The majority of our patients initiated high-efficacy DMTs already from disease onset and we showed that early exposure to high-efficacy DMTs had a protective effect and reduced the risk of future EDA-3 status. Conversely, similar protective effect was not observed for PIRA. However, the relatively low number of patients developing PIRA overall in the present cohort may be at least partially explained by the relatively high percentage of patients initiating high-efficacy DMT from disease onset.

We showed that high KFLC index at BL was independently associated with EDA-3 and PIRA. Since KFLC index is a measure of intrathecal free kappa light chains secreted by activated B lymphocytes in the CNS, our data suggest that these immunoglobulin-secreting cells play a role in the formation of focal inflammatory lesions, but probably also in neurodegenerative processes, increasingly recognized in RRMS as PIRA. The presence of leptomeningeal contrast enhancement is anatomically related to focal cortical thinning in MS [30], and interestingly such enhancement has been also associated with high KFLC index [11].

It has previously been demonstrated that innate immune cell activation (microglia/macrophages), assessed with PET, contributes to the diffuse tissue injury that is associated with PIRA [31]. In MS, B cells appear to demonstrate altered cytokine profiles, exhibiting increased interleukin (IL)-6 and granulocyte macrophage (GM)—colony stimulating factor secretion in comparison with healthy controls [32]. Cytokines such as IL-6 and GM-colony stimulating factor may potentially mediate inflammatory polarization of microglia, eventually resulting in microgliosis and tissue injury [33]. Thus, extensive and early B cell activation, reflected by high levels of intrathecal KFLC synthesis at BL, may be indirectly linked to microglial activation, with the ultimate consequence of PIRA.

In the present cohort, 18 out of 131 individuals (13.7%) developed PIRA during follow-up, which is in line with previous reports [16]. Importantly, as a consequence of the relatively short mean follow-up time of our study-cohort, the vast majority of patients with PIRA (17/18, 94.4%) exhibited “early” PIRA (i.e. within 5 years from onset), which has previously been demonstrated to be associated with worse prognosis during later stages of the disease [16]. Importantly, four out of 18 patients with PIRA exhibited new T2 lesions, indicating evidence of subclinical inflammatory disease activity. Such activity might be an important factor driving PIRA, particularly in the early stages of the disease. It would therefore be valuable to investigate the association of subclinical disease activity with PIRA in larger cohorts, as well as the association of intrathecal KFLC synthesis with progression independent of both relapse and MRI activity.

Other routinely measured CSF biomarkers included in our current analysis did not demonstrate any predictive value concerning EDA-3 and PIRA. Although, previous studies found associations between CSF NfL levels and long-term disability worsening and conversion to secondary progressive MS [34–36], all these studies had considerably longer follow-up times than the present study. CSF NfL seems to be a relatively weak predictor of conversion to CDMS from both radiologically isolated syndrome (RIS) and CIS [37, 38], and we could previously confirm that CSF NfL levels are moderate predictors of EDA-3 status within 24 months in patients with early RRMS [39]. However, in the present analysis, BL CSF NfL concentrations did not differ between EDA-3 and NEDA-3 status at follow-up. This is an unexpected finding, but in the present cohort, the predictive ability of CSF NfL might be mitigated by the relatively large percentage of patients receiving high-efficacy DMTs after the first demyelinating event. Although CSF levels of GFAP correlate with disability worsening [40, 41], BL levels of GFAP in the present study did not significantly differ between those who went on to develop PIRA and those who did not. Indeed, the majority of the patients included in the present cohort had low levels of CSF GFAP at the time of sampling according to age-adjusted reference intervals (median 330 ng/L, IQR 230 – 440) [25]. Nonetheless, blood levels of NfL and GFAP seem to be more promising in tracking disability worsening over time [42, 43], although some studies could not demonstrate an association of serum NfL with disability worsening [44]. Recently, the combined elevation of serum GFAP and NfL *z* scores was demonstrated to be associated with a 4- to fivefold increased risk of PIRA [43].

Also in other neurodegenerative conditions such as Alzheimer´s disease (AD), blood GFAP appears to be superior to CSF levels in terms of reflecting Aβ pathology, especially at the early stages of the AD continuum [45]. This finding is somewhat counterintuitive, as blood-based biomarkers of neurological conditions are generally considered to be an approximation of CSF levels. A potential reason for this is that astrocytes are part of the neurovascular unit that maintains the integrity of the blood–brain barrier which is commonly disrupted in MS [46]. Brain capillaries are known to be almost entirely covered by astrocytic end-feet [47], which regulate the dilation and constriction of microvessels [48]. Therefore, GFAP may thus be released directly from reactive astrocytes and into the circulation via this way.

One limitation of the present study is the relatively short follow-up time, and it is plausible that more individuals would go on to develop PIRA with an extended observational period. In our analysis, PIRA was defined according to the EDSS, and we did not have information on the Timed 25-Foot Walk Test and the 9-Hole Peg Test, which have previously been reported to influence PIRA [13]. As a result, we cannot exclude some degree of underestimation of PIRA in our cohort. Furthermore, we did not have information on retinal layer thickness as determined by optical coherence tomography which has recently been demonstrated to predict the risk of disability accrual in early RRMS, including PIRA [49].

In conclusion, we could demonstrate the robust prognostic ability of high KFLC index at BL in a real-world cohort of patients mostly exposed to high-efficacy DMTs already after the first demyelinating event. We show that high KFLC index is independently associated with a higher risk of EDA-3 and PIRA during follow-up. KFLC index has in recent years emerged as an exceedingly useful diagnostic biomarker with a high sensitivity and specificity for MS. Our data thus add important and clinically useful information to the growing body of knowledge regarding risk factors and prognostic predictors for EDA-3 and PIRA in MS. An expert group has recently concluded that determination of intrathecal KFLC synthesis should be considered for inclusion in the next revision of the McDonald criteria [50]. It is therefore highly plausible that determinations of KFLC index at BL will become an important part of the diagnostic as well as prognostic evaluation of patients investigated for suspected MS and may be useful for the guidance of treatment decisions.
